# The relationship between spousal support and quality of life in postmenopausal women: a cross-sectional study

**DOI:** 10.1590/1806-9282.20251068

**Published:** 2026-01-09

**Authors:** Sevinç Köse Tuncer, Papatya Karakurt, Selen Özdemir, Sonay Bilgin

**Affiliations:** 1 Erzincan Binali Yıldırım University, Faculty of Health Sciences, Department of Nursing – Erzincan, Turkey.; 2 Atatürk University, Faculty of Nursing, Department of Nursing – Erzurum, Turkey.

**Keywords:** Nursing, Menopause, Quality of life, Spouses, Women's health

## Abstract

**OBJECTIVE:**

In today's world, with the prolongation of human life span, a significant part of women's lives is spent in the menopausal period. Factors that negatively affect the health and quality of life of women experiencing this process constitute an important problem in terms of women's health. The aim of this study was to determine the relationship between spousal support and quality of life among menopausal women.

**METHODS:**

The cross-sectional study was conducted with 300 women aged 45–65 years, who were in menopausal period and volunteered to participate in the study, registered in four family health centers located in eastern Türkye. The data were collected using the "Personal Information Form," "Menopause Spousal Support Questionnaire," and "Menopause-Specific Quality of Life Questionnaire."

**RESULTS:**

The mean total score of the women on the spousal support questionnaire was 116.39±17.88. In the subdimensions, the highest score belonged to valuing support (41.35±7.21) and the lowest score belonged to sexual intimacy support (21.52±3.91). Quality of life questionnaire subfield mean scores were as follows: vasomotor 5.43±3.24, psychosocial 10.83±5.23, physical 17.84±13.03, and sexual 2.02±3.16. Spousal support and quality of life levels differed significantly according to demographic characteristics (p<0.05).

**CONCLUSION:**

Increased support from husbands decreases menopause-related complaints and improves quality of life. On the other hand, smoking, irregular exercise, not receiving hormone therapy, and a lack of information increase complaints. The results show that spousal support and healthy living habits are determinants of quality of life during menopause.

## INTRODUCTION

Menopause, which typically occurs between the ages of 45 and 55 years, is a natural and inevitable transition marked by the permanent cessation of menstruation due to loss of ovarian function^1^. However, it is often accompanied by a range of physical and psychological symptoms, including hot flashes, night sweats, headaches, vaginal dryness, decreased libido, irritability, and depression, which can significantly impair women's quality of life and affect their relationships with their spouses, families, and peers^2,3^. Recent research by Rodrigues et al. highlighted that many women experience fear and misinformation during the climacteric phase, often exacerbated by a lack of adequate support from healthcare professionals and their partners^4^.

In studies conducted in Turkey, it has been reported that women's domestic relations are strained during menopause, and they have problems with their spouses due to sexual reluctance, which negatively affects their lives. Support from a spouse was found to be more effective than other sources of social support^5^. However, most women state that they cannot receive sufficient support from their spouses during this period^6,7^. Studies have shown that as social support increases, menopausal symptoms decrease, and women approach this process more positively^6,8^. Therefore, including spouses in the process and raising their awareness can help women spend this period in a healthier manner. The menopause period constitutes an important part of life with the prolongation of women's lifespan. Healthy management during this period is of great importance for women's health. This study aimed to determine the relationship between spousal support and quality of life in menopausal women.

## METHODS

### Study design and participants

This was a cross-sectional study. The study population consisted of 1,154 menopausal women aged 45–65 years who were enrolled in four family health centers, which were determined by drawing lots by simple randomization from eight family health centers in the center of a province located in the east of Turkey. Although convenience sampling was employed, the selected family health centers were intentionally chosen from both urban and semi-urban settings with varying socioeconomic characteristics. According to regional data from the Turkish Statistical Institute (TUİK), the education and income levels of the participants broadly aligned with the demographic profiles of women aged 45–65 years in Eastern Turkey. This approach aimed to enhance sample representativeness despite the nonrandom selection of participants^9^. The sample was planned to consist of at least 293 individuals who applied to these centers between April and August 2024 and who met the inclusion criteria (being between the ages of 45–65, being in the menopausal period, having no communication and mental disability, and being literate) and 300 individuals were formed. The sample size was calculated using the formula proposed by Salant and Dillman^10^. Convenience sampling was used because of time limitations and ease of access to participants at selected health centers. However, the family health centers were located in socioeconomically diverse urban and semi-urban areas, which aimed to improve sample representativeness. Nonliterate women were excluded because the questionnaires (MSSQ and MENQOL) were self-administered and required reading comprehension skills. Including nonliterate women would have necessitated researcher assistance during data collection, potentially introducing response bias and compromising data reliability.

### Instruments

The researcher used the "Personal Information Form," "Menopause Spousal Support Questionnaire," and "Menopause-Specific Quality of Life Questionnaire" to collect research data.

**Personal Information Form:** The form developed by the researchers in line with the literature consisted of 12 questions on women's sociodemographic characteristics and menopausal information^11–14^.

**Menopause Spousal Support Questionnaire (MSSQ):** The scale developed by Idiana et al. and adapted into Turkish by Yavaş and Sümen consists of 17 items and 4 subdimensions (emotional, instrumental, caring, sexual intimacy) to measure the support perceived by menopausal women from their husbands^12,13^. Each item is scored between 1 and 10, with a total score ranging from 17 to 170. Higher scores indicate higher partner support. Cronbach's α value was found to be 0.84–0.96^12^ and 0.76–0.94 in this study.

**Menopause-Specific Quality of Life Questionnaire (MENQOL):** The Turkish validity and reliability of the scale developed by Hilditch et al. was conducted by Kharbouch and Şahin^14,15^. The scale comprises 4 domains and 29 statements. As the scores increase, the severity of complaints increases, and the quality of life decreases. The lowest and highest scores are 0 and 6, respectively. Subdomain scores are ranked from 0 to 6, with 0 indicating no problem, 1 indicating a mild problem, and 2–6 indicating severity. Cronbach's α value was found to be 0.73–0.84^14^ and 0.85–0.90 in this study.

### Intervention

The study data were collected by the researchers between April 2024 and August 2024 using the "Personal Information Form," "Menopause Spousal Support Questionnaire," and "Menopause-Specific Quality of Life Questionnaire," which were developed in line with the literature. Verbal consent was obtained from the women in the study. The data were collected using a face-to-face interview technique that lasted approximately 15–20 min.

### Statistical analysis

Data were analyzed using SPSS version 22.0. Descriptive statistics, such as frequency, percentage, mean, median, and standard deviation, were used. The conformity of the data to a normal distribution was examined using skewness and kurtosis analyses, and a range of ±2.0 was considered normal^16^. The t-test was used for two independent groups, and ANOVA was used for three or more groups. The difference between significant groups as a result of ANOVA was determined using the LSD or Dunnett's C test. The relationships between the variables were evaluated using multiple linear regression. Statistical significance was set at p<0.05. In addition to tests of statistical significance, effect sizes were calculated to assess the clinical relevance of the differences. Cohen's d was used for two-group comparisons, and η^2^ (eta squared) was used for comparisons involving multiple groups. According to conventional benchmarks, a Cohen's d of 0.20 is considered small, 0.50 medium, and above 0.80 large; for η^2^, values of 0.01, 0.06, and above 0.14 are interpreted as small, medium, and large effects, respectively. Reliability was measured using Cronbach's α.

## RESULTS

The mean age of the women participating in the study was 52.49±8.76 years. Of them, 39.0% were high school graduates, 50.6% were housewives, 53% perceived their income as equal to their expenses, and 15% exercised regularly. The mean age at menopause was 48.36±6.48 years. In addition, 28% had received and discontinued hormone therapy, 41% perceived menopause as partially affecting their lives, 37% perceived it as negatively affecting their lives, and 64% had received information on menopause (Table 1).

**Table 1:** Analysis of the relationship between Menopause Spousal Support Questionnaire, Menopause-Specific Quality of Life Questionnaire with sociodemographic and menopause variables.

	n	%	MENQOL				MSSQ
			Vasomotor	Psychosocial	Physical	Sexual	
			Mean±SD	Mean±SD	Mean±SD	Mean±SD	Mean±SD
**Educational status**							
Primary^a^	39	13.0	5.00±3.79	10.84±5.87	21.15±10.75	1.61±3.06	117.76±13.67
Secondary^b^	110	36.7	6.17±2.83	10.92±4.87	15.52±10.31	1.45±2.41	118.83±19.33
High School^c^	117	39.0	4.76±3.36	10.74±5.45	20.71±16.03	3.02±3.83	113.18±17.97
Bachelor's degree and above^d^	34	11.3	5.62±3.06	10.75±5.12	10.00±4.24	0.62±1.13	117.62±14.04
Test/p			F=4.094	F=0.026	F=7.333	F=7.442	F=2.138
			p=0.007*	p=0.994	p=0.000*	p=0.000*	p=0.096
			b>c		b, c>d	b, c>d	
			η^2^=0.040	η^2^=0.000	η^2^=0.069	η^2^=0.070	η^2^=0.021
**Working status**							
Retired^a^	37	12.4	6.65±3.75	10.77±4.89	19.66±12.15	2.33±3.36	123.67±16.52
Housewife^b^	152	50.6	6.03±2.95	11.42±4.44	19.66±14.03	2.55±3.54	116.11±17.51
Employed^c^	111	37	4.27±3.21	9.97±6.21	14.66±11.10	1.16±2.24	115.02±18.47
Test/p			F=12.392	F=2.566	F=5.153	F=6.770	F=2.605
			p=0.000*	p=0.079	p=0.006*	p=0.001*	p=0.076
			a, b>c		b>c	b>c	
			η^2^=0.077	η^2^=0.033	η^2^=0.043	η^2^=0.017	η^2^=0.001
**Income perception status**							
Income<expense^a^	96	32.0	5.59±3.14	10.96±4.36	18.53±12.41	2.31±3.24	112.15±15.45
Income=expense^b^	159	53.0	5.37±2.89	10.20±5.01	17.37±14.02	1.92±3.18	118.03±19.72
Income>expense^c^	45	15.0	5.26±4.48	12.73±7.04	18.0±10.68	1.73±2.94	119.60±14.17
Test/p			F=0.199	F=0.616	F=0.237	F=0.664	F=4.177
			p=0.820	p=0.616	p=0.789	p=0.515	p=0.016*
							b, c>a
			η^2^=0.004	η^2^=0.002	η^2^=0.004	η^2^=0.027	η^2^=0.037
**Hormone replacement therapy status**							
Not receiving treatment	216	72.0	5.86±3.24	10.70±5.29	19.56±13.96	2.44±3.42	113.87±18.17
Treated and then dropped out	84	28.0	3.50±.90	7.75±2.26	10.0±1.80	1.62±0.52	124.25±14.02
Test/p			t=6.909	t=3.967	t=8.824	t=10.495	t=-1.944
			p=0.000*	p=0.001*	p=0.000*	p=0.000*	p=0.029*
			d=0.97	d=0.69	d=0.87	d=0.38	d=-0.62
**Perception of menopausal period**							
Negatively affected^a^	111	37.0	6.24±3.30	12.27±5.67	28.89±12.74	3.67±3.97	111.35±12.24
Partially affected^b^	123	41.0	4.92±3.31	9.19±5.03	13.29±8.18	1.24±2.15	116.65±17.56
Not affected at all^c^	66	22.0	5.0±2.76	11.45±3.85	7.72±5.45	.68±1.72	124.36±23.03
Test/p			F=5.702	F=11.417	F=121.334	F=29.456	F=11.770
			p=0.004*	p=0.000*	p=0.000*	p=0.000*	p=0.000*
			a>b, c	a>b, c	a>b, c	a>b, c	b, c>a
			η^2^=0.037	η^2^=0.071	η^2^=0.449	η^2^=0.165	η^2^=0.073
**Status of getting information about menopause**							
Yes	192	64.0	4.97±3.49	9.66±5.67	14.30±14.79	2.88±3.82	119.17±19.42
No	108	36.0	5.68±3.08	11.48±4.86	24.20±10.31	1.53±2.61	111.44±13.48
Test/p			t=-1.837	t=-2.801	t=6.287	t=3.283	t=-3.666
			p=0.067	p=0.006*	p=0.000*	p=0.001*	p=0.000*
			d=-0.22	d=-0.34	d=-0.75	d=0.40	d=0.45

F: analysis of variance; t: independent groups t-test; η^2^: Eta^2^; d: Cohen's d; MSSQ: Menopausal Spousal Support Questionnaire; MEDQOL: Menopause-Specific Quality of Life Questionnaire; SD: standard deviation.

a Primary;

b Secondary;

c High school;

d Bachelor's degree and above. Letters indicate significant differences between groups according to LSD or Dunnett's C post-hoc test.

* indicates statistical significance at p<0.05. Bold values denote statistically significant findings (p<0.05).

Education, income perception, smoking, physical activity, hormone therapy, and menopause knowledge significantly affected spousal support and quality of life (p<0.05). Educated, employed, nonsmoking, active, informed women, and those who had received hormone therapy reported better quality of life and fewer complaints (Table 1). In addition to reporting p-values, this study included effect size calculations to assess the clinical relevance of the findings. Differences related to educational status and employment had small to medium effect sizes (η^2^=0.017–0.077). Although the differences in perceived income levels were statistically significant, the effect size was small (η^2^=0.027), indicating limited clinical significance (Table 1).

Conversely, the findings regarding perceptions of menopause revealed large effect sizes, particularly in the physical (η^2^=0.449) and sexual (η^2^=0.165) domains. Comparisons based on HRT use showed large effect sizes in the vasomotor (Cohen's d=0.97) and physical (Cohen's d=0.87) domains. When differences based on menopause-related knowledge were analyzed, a large effect size was observed in the physical domain (Cohen's d=-0.75), while moderate effect sizes were found in spousal support (MSSQ total score, Cohen's d=0.45) and sexual domain (Cohen's d=0.40) (Table 1). These results suggest that menopause perception, hormone therapy use, and knowledge acquisition are not only statistically significant but also clinically meaningful and strong determinants of quality of life among postmenopausal women.

The mean MSSQ score was 116.39±17.88. The highest subdimension was valuing support (41.35±7.21), and the lowest was instrumental support (18.42±3.62). On the MENQOL, the physical domain scored highest (17.84±13.03), and the sexual domain scored lowest (2.02±3.16) (Table 2).

**Table 2:** Mean scores of Menopause Spousal Support Questionnaire and Menopause-Specific Quality of Life Questionnaire instruments.

	Subdimension	Mean±SD	Min.	Max.
MSSQ	Emotional support	35.10±5.16	25	43
	Instrumental support	18.42±3.62	9	25
	Appraisal support	41.35±7.21	23	51
	Sexual intimacy support	21.52±3.91	12	28
	Total	116.39±17.88	75	142
MENQOL	Vasomotor	5.43±3.24	0	11
	Psychosocial	10.83±5.23	0	26
	Physical	17.84±13.03	0	56
	Sexual	2.02±3.16	0	11

SD: Standard deviation; Min: Minimum; Max: Maximum; MSSQ: Menopausal Spousal Support Questionnaire; MEDQOL: Menopause-Specific Quality of Life Questionnaire.

Regression analysis was conducted to examine the predictive relationship between spousal support (MSSQ total score) and menopause-related quality of life domains (MENQOL subdimensions), age, and age at menopause. In Model 1, which included only MSSQ total scores as predictors, higher spousal support significantly predicted fewer symptoms in the vasomotor (β=-0.319, p<0.001), psychosocial (β=-0.137, p=0.046), and physical domains (β=-0.232, p=0.001), but not in the sexual domain (p=0.473). The model explained 24.1% of the variance (R^2^=0.241), and the adjusted R^2^ was 0.230, indicating a moderate fit (Table 3).

**Table 3:** Simple Linear regression analysis of women's age and age at menopause with Menopause Spousal Support Questionnaire and Menopause-Specific Quality of Life Questionnaire.

	Model	Variable		B	SE	Β	t	p	95%CI		VIF
									Lower	Upper	
**MSSQ**	1	Constant		136.015	2.520		53.984	**0.000** *	131.057	140.974	
		MENQOL	Vasomotor	-1.756	0.385	-0.319	-4.564	**0.000** *	-2.514	-0.999	1.889
			Psychosocial	-0.467	0.233	-0.137	-2.007	**0.046** *	-0.925	-0.009	1.799
			Physical	-0.318	0.098	-0.232	-3.235	**0.001** *	-0.511	-0.124	1.993
			Sexual	0.316	0.44	0.056	0.718	0.473	-0.551	1.183	2.363
			R=0.490, R^2^ =0.241, Adjusted R^2^=0.230								
			F=23.362, Model (p)=**0.000** *								
			Durbin Watson=1.723								
**Age**	2	Constant		44.626	1.947		22.918	**0.000** *	40.794	48.458	
		MENQOL	Vasomotor	0.581	0.093	-0.474	-6.223	**0.000** *	0.397	0.764	2.033
			Psychosocial	-0.125	0.055	-0.164	-2.275	**0.024** *	-0.233	-0.017	1.824
			Physical	-0.032	0.023	-0.106	-1.379	0.169	-0.078	0.014	2.064
			Sexual	0.080	0.103	0.064	0.774	0.440	-0.123	0.283	2.368
			MSSQ	0.055	0.014	0.247	4.018	**0.000** *	0.028	0.082	1.317
			R=0.399, R^2^=0.159, Adjusted R^2^=0.145								
			F=11.150, Model (p)=**0.000** *								
			Durbin Watson=1.496								
**Average age of menopause**	3	Constant		39.645	1.865		21.255	**0.000** *	35.974	43.315	
		MENQOL	Vasomotor	0.547	0.089	-0.455	-6.116	**0.000** *	0.371	0.722	2.064
			Psychosocial	-0.110	0.053	-0.148	-2.098	**0.037** *	-0.314	-0.007	1.721
			Physical	-0.050	0.022	-0.168	-2.248	**0.025** *	-0.095	-0.006	2.102
			Sexual	-0.107	0.099	-0.087	-1.080	0.281	-0.302	0.088	2.268
			MSSQ	0.063	0.013	0.287	4.798	**0.000** *	0.037	0.088	1.417
			R=0.448, R^2^=0.200, Adjusted R^2^=0.187								
			F=14.737, Model (p)=**0.000** *								
			Durbin Watson=1.470								

SE: standard error; B: beta; β: standardized regression coefficient; t: independent groups t-test; R^2^: coefficient of determination; F: analysis of variance; MSSQ: Menopausal Spousal Support Questionnaire; MEDQOL: Menopause-Specific Quality of Life Questionnaire; CI: confidence interval; VIF: variance ınflation factor.

* indicates statistical significance at p<0.05. Bold values denote statistically significant findings (p<0.05).

In Model 2, the participants’ age was added to the model. Age positively predicted spousal support (β=0.247, p<0.001) and showed a significant inverse relationship with vasomotor (β=-0.474, p<0.001) and psychosocial symptoms (β=0.164, p=0.024). This model explained 15.9% of the variance (R^2^=0.159), with an adjusted R^2^ of 0.145 (Table 3).

In Model 3, age at menopause was included in the analysis. Later menopause age was significantly associated with higher spousal support (β=0.287, p<0.001) and reduced vasomotor (β=-0.455, p<0.001), psychosocial (β=-0.148, p=0.037), and physical symptoms (β=-0.168, p=0.025). This model had an R^2^ of 0.200 and an adjusted R^2^ of 0.187, indicating moderate explanatory power (Table 3).

Across all models, the adjusted R^2^ values were slightly lower than the R^2^, as expected, but still demonstrated consistent model robustness. The findings emphasize that increased spousal support and later onset of menopause are associated with improved quality of life across multiple menopausal domains.

## DISCUSSION

In this study, the sociodemographic characteristics, lifestyle factors, perception of menopause, level of spousal support, and quality of life of women in the menopausal period were evaluated. The participants’ average age was 52.49 years, and the average age at menopause was 48.36 years. The fact that more than half of the women were housewives and only 15% exercised regularly aligns with the sociocultural structure of the region where the study was conducted. When the demographic characteristics of the participants were compared with the 2024 TÜİK^9^, the average age of women in our study (52.49 years) was consistent with the regional average age for women aged 45–59 years (52.3 years). The rate of housewives in our study was 54.7%, whereas the TÜİK data reported 51.2%^9^. The rate of high school and higher education in our study was 36.7%, compared to 33.5% in the TÜİK data^9^. These results indicate that our sample generally reflected the regional sociodemographic structure.

The WHO emphasizes that physical activity during menopause is critically important for symptom management and cardiometabolic health^1^. Both in Turkey and internationally, it has been reported that low levels of physical activity negatively affect quality of life by increasing symptoms such as vasomotor complaints, sleep disturbances, and depressive symptoms^2,3,17^. In our study, women with higher levels of physical activity had higher average MENQOL and MSSQ scores, suggesting that an active lifestyle can strengthen not only personal well-being but also family relationships. The study found that educational status and employment had a statistically significant effect on quality of life, albeit with small to moderate effect sizes, with higher average MSSQ scores observed among women with higher education and those who were employed. Literature indicates that higher education increases women's awareness of menopausal symptoms, facilitates access to health services, and strengthens coping skills^11,18,19^. The limited impact of perceived income suggests that quality of life is influenced not only by economic status, but also by social support, cultural norms, and individual coping strategies.

The study found that both HRT use and knowledge about menopause were significantly associated with quality of life and spousal support. The findings show that in women using HRT, there were especially large effect sizes in the vasomotor and physical domains; this is consistent with the literature reporting that HRT can significantly reduce symptom burden in suitable cases^4,20^. Knowledge of menopause not only facilitates symptom management but also strengthens communication with one's spouse and increases perceived social support. Indeed, Baral and Kaphle^20^ emphasized in their study that a lack of knowledge about menopause can lead to incorrect treatment choices and unnecessary anxiety, whereas correct information strengthens social support relationships. Additionally, studies highlighting the role of melatonin and thyroid function in the management of menopausal symptoms indicate that hormonal and endocrine approaches should be considered from a multifaceted perspective^17^.

The results of this study indicate that cognitive function is a critical aspect of quality of life during menopause. Zangirolami-Raimundo et al.^21^ reported that the cognitive performance of postmenopausal women is related to their daily living activities and technology use skills. In this context, it is important to plan supportive interventions in menopause management that target the physical, psychosocial, and cognitive domains. In this study, the perception of menopause emerged as a significant determinant of both quality of life and spousal support. Women who viewed menopause as a natural process had higher average MSSQ and MENQOL scores. Polat and Karasu^5^ reported that a positive perception of menopause strengthens social relationships by enhancing coping skills, whereas a negative perception increases both psychological and physical burdens. Similarly, Cronin et al.^22^ emphasized that managing menopausal symptoms with digital health technologies can particularly support psychosocial well-being in the workplace.

The most striking finding of this study was the positive relationship between spousal support and quality of life. Women with high spousal support during menopause had significantly lower mean scores for vasomotor, psychosocial, and physical symptoms, and their quality of life increased accordingly. International studies have shown that the knowledge and support men provide about menopause are key determinants of women's quality of life^9,23,24^. Qualitative research in Turkey indicates that, especially under the influence of traditional family structures, spouses do not provide adequate support in this regard; women often have frequent arguments with their husbands, and this negatively affects both the severity of menopausal symptoms and quality of life, as well as marital harmony^5,25^. These findings indicate that men's knowledge of menopause and spousal support during menopause is insufficient in Turkey. Our study underscores the need to develop women's health policies that reduce the negative impact of cultural norms on women's health. In Brazil, national women's health policies have been shown to reduce health inequalities during menopause^19^. In China, the active participation of husbands in the menopause process lowers women's symptom scores^23^, while a study from Saudi Arabia reported that social support is inversely related to the severity of menopausal symptoms^26^. These data clearly indicate that in collectivist societies (such as the Eastern and Southeastern Anatolia regions of Turkey, China, Japan, and India), spousal support can play a central role in menopause management.

In our study, we found that as women's age and age at menopause increased, vasomotor, physical, and psychosocial complaints increased; conversely, spousal support was found to reduce these complaints. The literature also reports that age is a significant determinant of menopausal symptoms^2,20^. Furthermore, spousal support alleviates the increasing symptom burden that comes with age during menopause and improves quality of life^6,24^. These findings demonstrate that age increases symptom severity, whereas spousal support mitigates this negative effect.

## CONCLUSION

This study revealed that the quality of life of postmenopausal women living in the Eastern Anatolia region is significantly associated with sociodemographic factors, lifestyle characteristics, perception of menopause, level of knowledge, use of hormone therapy, and spousal support. The findings indicate that spousal support is not only an emotional resource but can also contribute to physiological well-being by reducing the burden of vasomotor, psychosocial, and physical symptoms.

The study found that as the level of support women receive from their spouses during menopause increases, complaints in the vasomotor, psychosocial, and physical domains decrease. In line with this finding, husbands should be actively involved in the care processes of women receiving healthcare during menopause, and communication and interaction between spouses should be strengthened. By providing men with sufficient information and emotional support regarding the menopausal period, the level of support they offer their wives can be increased, thus enabling women to navigate this process more smoothly.

The findings suggest that community-based interventions aimed at strengthening spousal support during menopause could be an important strategy for maintaining and improving quality of life. Future longitudinal and/or mixed-methods studies are expected to more clearly reveal the causal aspects of the relationship between spousal support and quality of life, as well as the role of the cultural context. Additionally, developing educational programs to increase spouses’ knowledge of the menopausal process may provide benefits at both the individual and societal levels.

## Data Availability

The datasets generated and/or analyzed during the current study are available from the corresponding author upon reasonable request.
